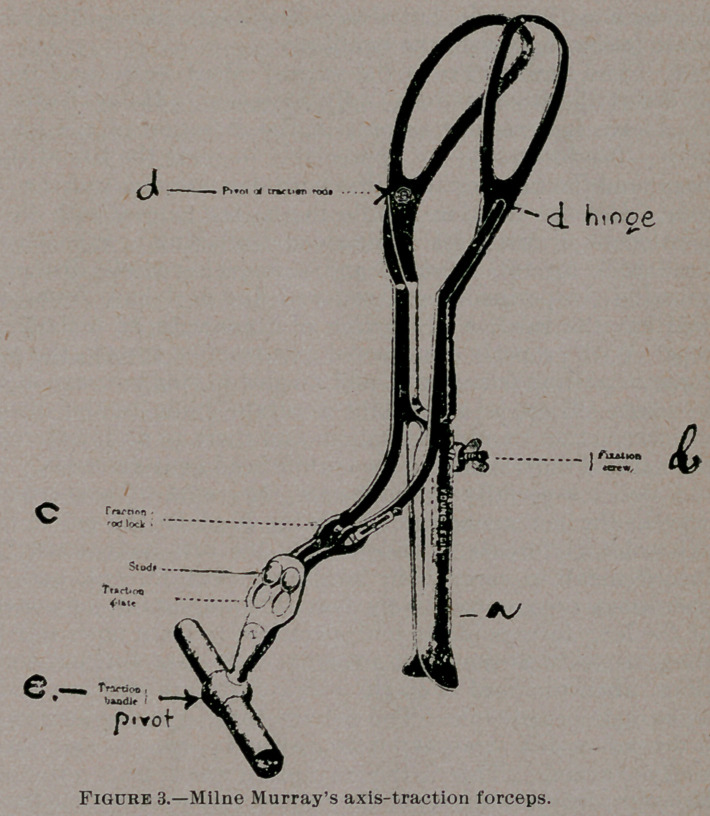# On Axis-Traction Forceps*Read at the Thirty-third Annual Meeting of the Texas State Medical Association, Galveston, April 25, 1901.

**Published:** 1901-07

**Authors:** William Keiller

**Affiliations:** F. R. C. S. (Ed.), Professor of Anatomy, Medical Department, University of Galveston, Texas


### TEXAS MEDICAL JOURNAL.

ESTABLISHED JULY, 1885.

PUBLISHED MONTHLY.—SUBSCRIPTION $1.00 A YEAR.

VOL. XVII. AUSTIN, JULY, 1901. No. 1.

### Original Contributions.

For Texas Medical Journal.

### On Axis-Traction Forceps.

BY WILLIAM KEILLER, F. R. C. S. (ED.),
Professor of Anatomy, Medical Department, University of Galveston, Texas.

GENTLEMEN: When I heard that you had appointed me Chairman of the Section on Obstetrics of this Association my first thought was one of pleasure because of the honor you had conferred upon me, my second a realization of my unfitness to fill satisfactorily such an honorable position in a branch of medical practice in which I have to confess to a somewhat limited experience during the ten years that I have spent among you.

As Chairman of this Section in the year 1901 it would not be out of place were I to glance briefly at the enormous progress that has been made in obstetrics in the past century, or, indeed, in the past twenty years; and in such a retrospect one would have, among other things, to allude to the present status of forceps delivery, as one of the most important of life saving devices, which has at least been thoroughly legitimized and brought almost to perfection during the century just gone. Not second in importance, but of much later birth, would be antiseptics and the almost complete exclusion from all up to date practice of the deadly home-desolating puerperal fever. And then would come the whole rational physiology and pathology of pregnancy and labor, and most of obstetric surgery.

Today the physician who has a case of puerperal fever develop where he has been in full charge from the beginning should have,

*Read at the Thirty-third Annual Meeting of the Texas State Medical Association, Galveston, April 25, 1901.

to say the least of it, a very uneasy conscience, and in city practice Cæsarean section should be so safe as to make craniotomy a crime.

I propose, however, to ask your consideration of axis traction forceps, the more so that the teaching of the Edinburgh school and my practice for the past twelve years has been different from the teaching of American text-books. American text-books recommend axis traction forceps only when the head of the child is entering the pelvic brim, while the Edinburgh school uses no other forceps for any position, considers them the best possible instruments in all cases where forceps are indicated, and some regard them as an excellent means of saving the perineum.

By way of retrospect, in the early part of the past century forceps were dreaded, too often meant the death of both the mother and child, and the legitimacy of their use was hotly contested by a large section of the profession. One does not wonder that it is so, when one learns that they were often covered with leather to make them softer and less formidable in appearance, were taken surreptitiously out of the dirty pocket of the accoucheur, slipped in stealth under the bed clothes, and applied with dirty hands through the unwashed vulva, without if possible letting the friends, patient or even the nurse know they were being applied.

Today if a woman is once delivered by forceps it is difficult to get her to have patience to wait on nature in any subsequent labor, it is often the patient and her anxious friends rather than the physician who urge their use; the instruments are sterilized by boiling, the patient carefully cleansed, the pain abolished by an anesthetic, the instruments used with all the precautions of modern surgery, and the operation one of the safest of modern procedures, if not absolutely devoid of danger.

Let us first glance at the mechanism and principles of axis traction. The head of the child must descend through a curved canal whose axis is ever changing, while the head itself must all the time be free to change its position through various phases of increase of flexion, internal rotation, extension and restitution. If the expulsive force of the uterus is to be substituted by mechanical traction an ideal tractor should grasp the head firmly with the least possible compression, and should be fixed as near the center round which the head bends and rotates as possible. It should offer no resistance to the necessary movements of the child's head, should make traction possible in the axis of the part of the canal in which the head lies, should give the greatest possible result with the least possible expenditure of force, and should indicate in all possible stages of the descent the exact direction in which traction should be applied.

All this is done with almost perfect accuracy by properly made axis traction forceps. It is evident that a straight pull on the handles of the ordinary forceps as in Fig. 1 will result in much waste of force in pulling the head of the child against the pubes of the mother, and only a fraction of the force expended will act in the direction of the pelvis axis. This defect is overcome by a down-

FIGURE 1.—To illustrate how traction on the handle at A will waste much force against the pubes of the mother at B.

ward and backward pull given with the left hand, and a forward leverage by the right hand.

At how much disadvantage does the obstetrician work; and who shall say whether he is levering or pulling in the right direction? Besides this, flexion and internal rotation of the head high up, and extension as the head passes out of the lower segment are impeded if not prevented by the rigid instruments.

Now let us see how the axis-traction forceps clear up all this. I describe the action of the Milne-Murray-Tarnier forceps which I use myself, or the Jewett adaptation of Milne-Murray's principles in the Jewett forceps, which I show you, and which is the American instrument which approaches nearest to the favorite Scotch pattern.

With the instrument in figures 2 and 3, the necessary conditions are met as follows:

1. The blades are adapted to the average pelvic curve, and grasp

FIGURE 2.—The traction rods will apply the full force in the line d e, which is the axis of that segment of the pelvis through which the head is descending.

the head firmly without much pressure. The handles (Fig. 3a) are simply used for applying the forceps and holding them in place; the forceps are fixed with the thumb screw (Fig. 3b) which is never made tighter than is just necessary to keep them accurately adjusted. After the instruments are on, the handles should never be touched. They further become, as I shall show, a most accurate index of the direction in which the accoucheur should pull.

2. The traction rods (Fig. 3c) are hinged at *d*, not it is true in the center of the head, but as near that center as is practicable. They in no way interfere with the flexion or extension of the child's head, and a swivel at *e* allows the head to rotate freely.

3. The rods are rigid and bent till the line of traction (Fig. 2de) in the axis of the segment of the pelvis through which the head is passing. All force used in pulling on the handles at (Fig.

FIGURE 3.—Milne Murray's axis-traction forceps.

2e) will be exerted exactly in the line *de* which is the best possible direction for the descent of the head through the part of the pelvis which it at present occupies.

4. As the head descends it will, if the instruments have been applied at the pelvic brim, first become more flexed and the hinge at *d* will permit of this. Such flexion will carry the handles further backward, and this will not be interfered with if the rods be kept just clear of the handles; thus the line of traction must be at this stage shifted a little backward. With this increase of flexion, internal rotation will also go on, and will be permitted by the pivot *e*, and in the high operation it will be found necessary to take off the forceps when the head is well down in the pelvis and reapply them in the pelvic curve.

5. Now with the head well engaged in the pelvis the axis of the canal is continually changing. Nothing prevents the head changing axis; and more than that, as the head bends forwards the handles move forward with it, and act as a perfect index to the direction in which traction should be applied. The obstetrician has only to keep his traction rods parallel with the handles and he can not possibly pull in the wrong direction. The only mistake he is liable to make is a tendency to push the rods against the handles; this he must never do, but always keep them just a little clear. He must follow, not push, the handles.

6. And now we are at the vulva. Shall we remove the forceps or desert the traction rods? By no means. Here, if ever, the utmost care is necessary to pull in the direction of the least resistance. The forceps do not appreciably increase the size of the child's head, and the handles are still a most delicate index of the changing axis. Use the traction handle to the last; draw with the utmost gentleness, drawing for a minute and relaxing or even pushing the head back for two minutes, and so on, alternately distending and relaxing the perineum and you will find as I have found that you will have as small a proportion of tears with as without the forceps. With properly constructed axis-traction forceps the minimum force in pulling is required, and no side to side leverage at all must be used. There is a constant accurate guide to the direction in which traction should be applied, and there is no interference with the natural movements of the child's head as it descends.

I desire especially to emphasize the value of axis-traction forceps in the low operation. With the head on the pelvic floor, the axis-traction forceps is undoubtedly the best instrument; for it is inconvenient to carry both long and short forceps, and, as high operations are comparatively few, it is important, by constant use of the long forceps in the low operation, to train one's self to apply them as easily as the short straight ones. But further, the indications, when the head is on the pelvic floor, are still best met by the axis-traction principle. The pelvic canal from the coccyx forward is by no means straight; it is distinctly curved, and the axis is always varying. This is the very place where tears are most likely to occur; it is, therefore, of the utmost importance that the head be borne in its smallest diameters. In unassisted labor the head descends with its smallest suboccipito-bregmatic diameter in the dilating ring of the maternal tissues, and the chin flexed on the sternum, till the occiput engages under the symphysis; then the suboccipital region is caught by the pubic rami, and brow and face rotate round this point. Thus an ever-increasing diameter of the head passes over the perineum; first suboccipito-bregmatic, smallest of all, then the suboccipito-frontal, suboccipito-facial, and lastly the large suboccipito-mental; and if the perineum does not split it is a wonder.

Here at least it is the duty of every physician to assist nature, to prevent extension of the head, and to secure that instead of becoming fixed under the pubic arch, the occiput of the child shall continue to descend with the rest of the head, and that the extension shall not be allowed to take place till after the perineum has been passed. This can be done, when the forceps is not in use, by grasping the head thus: The thumb in the distended anus catching the face, and the fingers over the occiput, so that the whole head is under complete control and its extension can be prevented; while the fingers over the occipital region can drag the occiput down and encourage descent of the whole child with the head in the flexed position.

Where the forceps is used, let it be still used as a true axis-traction instrument; by no means forsaking the rods and taking to the handles, as is advised by the Tarnier school. Be more careful than ever to follow the indications as to the axis of traction given by the handles, and get the head born by very gentle traction between the pains.

Thus Milne-Murray stated ten years ago, that in ten years practice, where he has used axis-traction forceps in all kinds of cases, he has never had a single tear beyond the slight slit in the margin of the mucous membrane which is inevitable in all first labors.

Personally, I have had very good results. Croom especially emphasizes the use of this forceps in saving the perineum in occipito-posterior presentations.

Passing now to the high operation, if axis-traction forceps are of value in the low operation they are invaluable when the head is at the brim. Milne-Murray states emphatically that "a properly constructed pair of axis-traction forceps will deliver a child in a flat pelvis down to the limits which can be dealt with by turning, and with a much greater chance of delivering it alive." I can myself speak of the comparative ease with which a full-time child can be delivered through a true conjugate of three inches. Milne-Murray has delivered a full-time child through a true conjugate of 2.75 inches.

It is a mistake to state that in such cases the occipito-facial grasp that one gets of the head when it is lying in the transverse at the brim causes bulging in the biparietal diameter; lengthening occurs, not transverse bulging. Further, the tendency of the forceps to favor rotation downward of the anterior parietal bone is of the utmost importance in facilitating the progress of labor in flat pelves, this being an essential point in the mechanism of these cases. The success of turning mainly depends on when it is undertaken; after the uterus is drained of fluid it is always more hazardous; and if the accoucheur is to turn with safety to the child he must diagnose the case early, and turn as soon as the os is sufficiently dilated, if necessary breaking the membranes to do so, or using the bipolar method. I have no hesitation in saying that, with a true conjugate at the brim of three inches and a dry uterus, axis-traction forceps will give a much better prognosis as regards the child, and no worse as regards the mother.

Lastly, let me allude to the value of axis-traction forceps in hastening delivery of the breech. With the breech in the cavity or at the pelvic floor, I can speak from experience of the efficient manner in which they hold, whether applied over the sacrum and flexor surface of the thigh or over the outside of both thighs.

Of course, as always, traction is to be applied gently.

By the kindness of the McDermott Instrument Co., of New Orleans, La., I have several varieties of axis-traction forceps which I beg to present to you. The Jewett forceps is constructed according to specifications laid down by Milne Murray, the man who has put the construction of these instruments on a scientific basis. It is light, easily applied, and correctly constructed, and equally suitable for high or low operations.

I cannot see any use for the outward bend of the handles and the fixing screw is unnecessarily long.

The Tarnier forceps is too heavy for low operations, unnecessarily heavy for all operations. I do not think the cephalic curve is so correct as in the Simpson or Jewett type, nor the pelvic curve either, for that matter; but in France the forceps is applied with reference to the head of the child, not the pelvic curve.

Lusk's forceps is only suitable for the high operation; and is unnecessarily clumsy, and the handles and traction bar interfere with each other. I do not think the line of traction is in the proper axis, or that they fulfill the axis-traction principle.

The movable traction rods designed to be hooked on to the fenestra of any pair of long forceps carry out the axis-traction principle fairly well; but their liability to detachment between whiles when the traction intermits, and the necessity of attaching them after the forceps are applied or investigating their attachment occasionally to see that they are all right, and the further necessity of having a fixing screw to keep the forceps in place applied to any forceps with which they are to be used, make them infinitely inferior to properly made axis-traction forceps.

## Figures and Tables

**Figure 1. f1:**
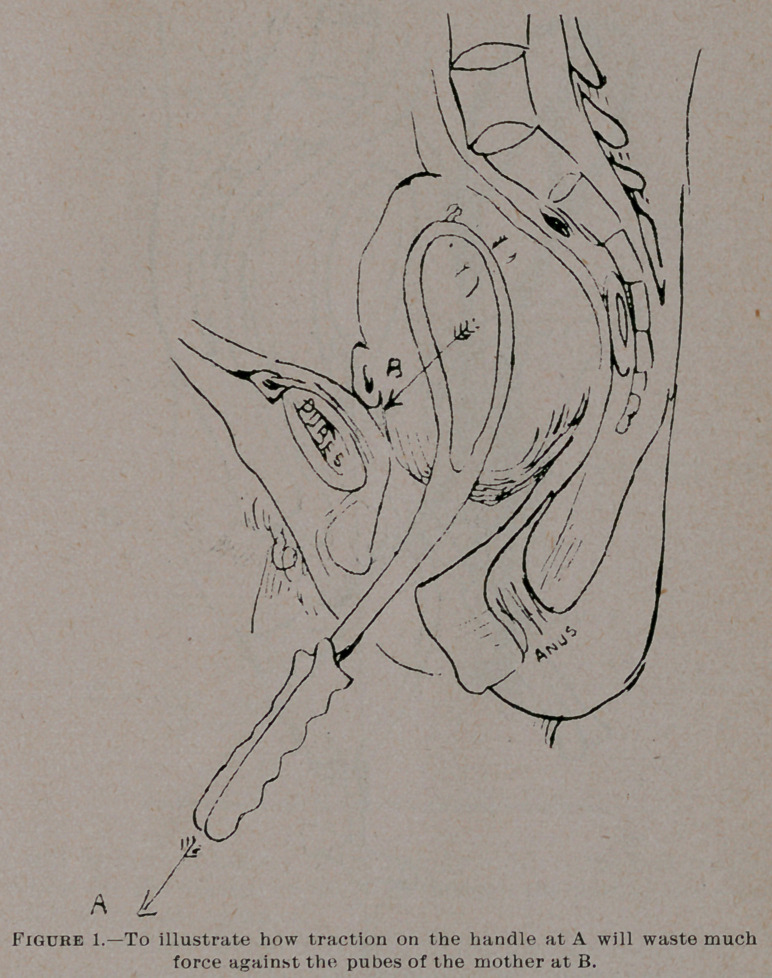


**Figure 2. f2:**
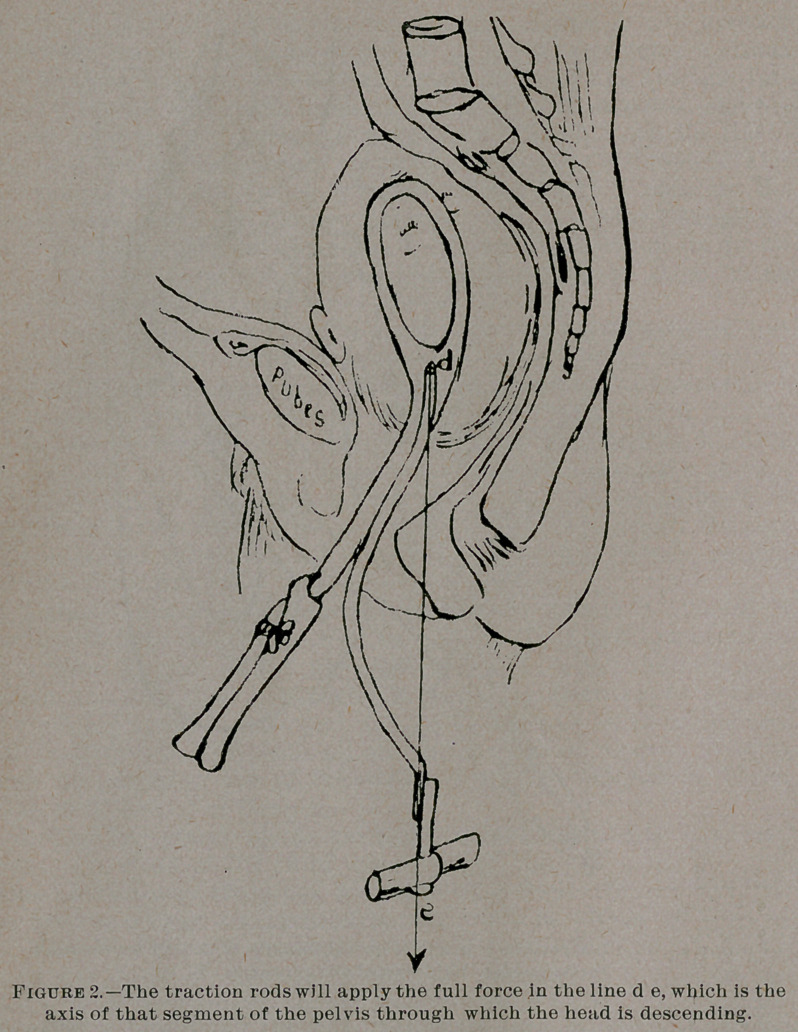


**Figure 3. f3:**